# The privilege of COVID‐19 vaccine wastage ‐ Hong Kong

**DOI:** 10.1002/puh2.39

**Published:** 2022-11-14

**Authors:** Joshua Yeuk Shun Tran, Phoebe Chi Fei Chan, Samuel Yeung‐shan Wong, Martin Chi‐sang Wong

**Affiliations:** ^1^ Faculty of Medicine The Chinese University of Hong Kong Hong Kong SAR China; ^2^ Li Ka Shing Faculty of Medicine University of Hong Kong Hong Kong SAR China; ^3^ JC School of Public Health and Primary Care Faculty of Medicine The Chinese University of Hong Kong Hong Kong SAR China; ^4^ Centre for Health Education and Health Promotion JC School of Public Health and Primary Care Faculty of Medicine The Chinese University of Hong Kong Hong Kong SAR China

**Keywords:** COVID‐19, vaccine inequity, vaccine wastage

## Abstract

COVID‐19 and its effects continue to ripple through Hong Kong. From strict social restrictions to loosening travel restrictions, from high death rates to high caseloads, and from low vaccine uptake to high vaccine uptake, Hong Kong has experienced the spectrum of COVID‐19. Hong Kong being in a position of privilege was able to procure at least three doses per person soon after vaccines became available. However, the public's response to the vaccines was suboptimal, with less than 55% of the population being vaccinated 6 months after the introduction of the vaccines. Though the government tried to encourage active vaccine uptake, various factors, such as perceived barriers, risks, and general distrust, contributed to the hesitancy experienced by the population. Logistical factors such as open and closed vial wastage may also contribute to vaccine waste. This may have led to massive vaccine waste of up to 2 million doses and more uncertainty with how to manage with to‐be‐delivered vaccines. Vaccine nationalism opened the doors to potential wastage, a symptom of vaccine inequity. Urgent action is needed to address uptake hesitancy and potential suboptimal logistical management to prevent further vaccine wastage.

After five COVID‐19 waves, almost 1.5 million confirmed cases, and over 9000 deaths [], Hong Kong continues to face challenges in its battle against COVID‐19 with over 7000 daily cases as of August, 2022. The cases in Hong Kong have been cyclical, with periods of high case loads that are suppressed by corresponding restrictions and precautions differing from peak to peak. Though Hong Kong is now quickly approaching one of the highest daily cases since its first case on 23 January, 2020, the city's restrictions are the most relaxed compared to previous peaks, due to its high vaccination rates. As of August 2022, 93.3% of Hong Kong's population have received ≥1 dose, 90.3% have received ≥2 doses, and 71.0% have received ≥3 doses [1]. While masks are still mandatory, all venue types (such as swimming pools, bars, and karaoke venues) are open to the public. The government has further relaxed the quarantine restrictions for inbound travelers from 7 days mandatory hotel quarantine to 3 days hotel quarantine with a further 4 days observation period. However, Hong Kong paradoxically has high death rates, especially during the fifth wave (6 January, 2022–21 March, 2022) reaching up to 37.7 deaths per million population [2]. One contributing factor to the relatively high death rates was a low vaccination rate prior to the fifth wave despite the early COVID‐19 vaccine introduction. While the Pfizer‐BioNTech and Sinovac vaccines were introduced in February 2021, uptake rate stagnated with only 67% having received ≥1 dose, 64% having received ≥2 doses, and only 5% having received a third dose up until December, 2021 [2].

Figure 1 shows the proportion of population vaccinated in Hong Kong with a completed initial protocol (two doses for Sinovac or Pfizer‐BioNTech), or partially completed initial protocol (1 dose of either vaccine) on May 24th, 2021. This time point was selected as government figures for the number of unused vaccines were published around this date. Vaccines initially became publicly available in Hong Kong from February 24, 2021, but two months later, the uptake rate was slow relative to other comparable high‐income countries’ vaccination rates at the same time point. A portion of the discrepancy can be attributed to the lag in vaccine purchasing and availability across different countries, but there stands a drastic difference of 17% with any doses in Hong Kong, compared to other high‐income countries (HIC) with double the average proportion.

**FIGURE 1:**
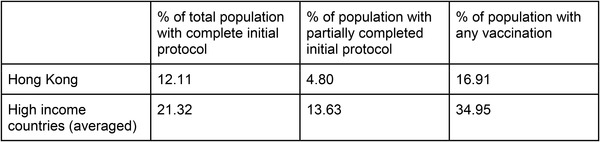
Proportion completed and partially completed initial protocol of COVID‐19 vaccines by May 24, 2021[4]

In December 2020, 7.5 million doses each of Sinovac, Pfizer‐BioNTech, and AstraZeneca were purchased via Advanced Procurement Agreements (APA). However, after the rollout of the initial 2 million doses each of Sinovac and Pfizer‐BioNTech, the government quickly recognized that receiving the AstraZeneca shipment would lead to certain stockpiling and wastage as over 1.05 million Sinovac vaccines and 840,000 Pfizer‐BioNTech vaccines doses expiring in mid‐August 2021 were reported to be unused [3]. The government subsequently discussed delaying the delivery of 5.5 million doses each of Sinovac and Pfizer‐BioNTech in August, 2021 and donated the ordered 7.5 million doses of AZ to the COVAX Facility in October, 2021. However, this does not eliminate the possibility that the reported unused vaccines were going to waste due to the late adoption of vaccinations amongst the Hong Kong population and the potential suboptimal logistical management.

## Vaccine wastage ‐ A symptom of vaccine inequity

Vaccine wastage refers to an oversupply of vaccines for a population which has low uptake leading to an unused surplus. Vaccine inequity persists despite over 1.5 years since the development of the first COVID‐19 vaccines [5]. Hong Kong's APA was in violation of the WHO's Fair Allocation Framework [5], which recommended that initial allocation be proportional to vaccinating 20% of every country's population. The opportunity for wastage only arose when Hong Kong instead displayed vaccine nationalism in their direct purchase agreement with pharmaceutical companies, ordering 0.5 times more doses than needed to vaccinate 100% of the population. However, vaccine wastage can still occur even if vaccine access is “equitable.” Initiatives such as COVAX and HIC donating about‐to‐expire vaccines to attempt to increase vaccination rates in lower‐middle‐income countries (LMIC) only impose more logistical challenges by shortening rollout time. Strengthening vaccine infrastructure, such as cold chains, is needed alongside equitable vaccine distribution to prevent furthering vaccine inequity.

## Contributing factors to COVID‐19 vaccine wastage

Vaccine wastage is affected by supply and uptake factors. Uptake is consistently negatively correlated with concerns over safety of the vaccine [6, 7]. The perception of vaccine safety is likely a cumulation of many factors, such as lack of confidence in newer vaccine mechanisms (mRNA), new pharmaceutical manufacturers, side effects [6], and the short timeframe (under 1 year) in which the vaccine was developed. The hesitancy toward vaccinations may be difficult to address effectively in a short period of time, particularly taking into account that the normal process to develop a viable human vaccine averages about 10 years. The developing vaccine is seemingly rushed through the approval process with “emergency use” authorizations in multiple points. A general uncertain sentiment towards the vaccines in Hong Kong is not surprising, especially regarding long term side effects.

Perceived barriers to accessing the vaccines, such as lockdowns and difficulty in getting vaccination appointments also pose a negative correlation [7]. Due to the effectiveness of the early 2020 strict social restrictions such as mandatory universal masking, closure of borders, closure of most venue‐types, and limits on the number of people per group in public in managing outbreaks, perceived susceptibility of the disease was low. Combined with the perceived risks of adverse effects of the vaccine and access barriers, these factors outweighed the perceived benefits, leading to a decrease in COVID‐19 vaccine uptake.

On the other hand, there are factors that positively correlate with vaccine uptake. Confidence in the government [8] among other factors, has been shown to increase acceptance. However, some of these factors may be affected by the local social environment. There was evident declining trust towards the government after the onset of the Anti‐Extradition Bill in June 2019, leading to certain subpopulations’ doubt regarding the pandemic response policies the Hong Kong government enacted [9]. The lack of trust in the government causes difficulty in public acceptance of government‐imposed policies, which includes encouraging vaccine uptake.

Interestingly, vaccine hesitancy has been a longstanding issue within Hong Kong healthcare workers. This was repeatedly seen when new flu strains were incorporated into the flu vaccine. In 2005, healthcare workers’ intention to take the new H3N2 Switzerland strain vaccine was 31.8% while in 2009, the intention to take the new H1N1 strain vaccine was 47.9% [10]. The divided opinions among healthcare workers likely affected the public's perception about the efficacy and safety of the newly introduced vaccines. Healthcare workers’ acceptance is an important affirmation to the public, perhaps even more so during controversial periods.

Apart from vaccine hesitancy, the logistical management of the vaccines may also contribute to wastage. Broadly speaking, there are two main types of vaccine waste, closed and open vial [11]. Closed vial includes issues such as loss of vaccine integrity and vaccine expiry. Hong Kong's wastage can be mainly attributed to closed vial vaccine expiry due to low uptake. This does not, however, dismiss the possibility of open vial wastage, such as insufficient demand of already opened vaccines [11]. Vaccine waste may easily occur when patients miss vaccine appointments or when a new 5‐dose vial is opened for less than five appointments.

The fifth wave, as the most persistently severe, likely served as a major motivator towards vaccine uptake. Within 6 months, the uptake rates increased to 87.51% having completed the initial protocol with an additional 3.18% having been vaccinated with one dose [4]. This ranks Hong Kong amongst the highest global vaccination rates.

## Recommendations for the Hong Kong Government

In light of Hong Kong's current high vaccination rate, there are some practical actions Hong Kong could have undertaken and can still undertake to reduce vaccine wastage [11]. First, to provide detailed transparent data on COVID‐19 vaccine wastage and the handling of the wastage. Potential unreported wastage from logistical mismanagement of vaccine transport and administration can be identified and addressed accordingly with specific and targeted solutions. Second, the government should accurately assess the public's acceptance of vaccination prior to procuring vaccines and to accurately educate the public. This strives to correct the local unbalanced supply and demand for vaccines. Though impossible to do perfectly, it allows for the government to grab hold of the general sentiment when making estimations. Third, the government should establish a plan incase of vaccine surplus, in order to avoid situations like near‐expiry donations from HIC wastage to LMICs that become unusable due to logistical limitations on the timeline.

## CONCLUSION

Vaccine wastage is a global issue, but the responsibility lies within individual healthcare systems to manage both supply and uptake. Access to vaccines is a public health necessity and right, but in the midst of global vaccine inequity, vaccine wastage is a privilege that the global health community cannot afford. Hong Kong has shown different factors that led up to its potential massive wastage, from vaccine hesitancy to over‐purchasing and poor contingency management plans for vaccine surplus. One can only hope for lessons to be learned for other governments that share this same privilege and duty to combat vaccine wastage.

## AUTHOR CONTRIBUTIONS

Joshua Tran: Conceptualization, Investigation, Writing ‐ Original Draft, Writing ‐ Review & Editing, Visualization, Supervision, Project Administration. Phoebe Chan: Conceptualization, Investigation, Writing ‐ Original Draft, Writing ‐ Review & Editing. Samuel Wong: Validation, Writing ‐ Review & Editing. Martin Wrong: Validation, Writing ‐ Review & Editing.

## CONFLICT OF INTEREST

Dr. Martin Chi‐sang Wong is an Editorial Board member of Public Health Challenges and a co‐author of this article. To minimize bias, he has been excluded from all editorial decision‐making related to the acceptance of this article for publication.

### ETHICS STATEMENT

Ethics approval is not applicable for this art.

## Data Availability

All data relevant to the study are included in the article.
